# Cerebrospinal fluid T1 value phantom reproduction at scan room temperature

**DOI:** 10.1002/acm2.12659

**Published:** 2019-06-09

**Authors:** Akihiro Yamashiro, Masato Kobayashi, Takaaki Saito

**Affiliations:** ^1^ Department of Radiology Nagano Red Cross Hospital Nagano‐City Nagano‐ken Japan; ^2^ Department of Radiology Shinano Town Shin‐Etsu Hospital Kamiminochi‐gun Nagano‐ken Japan; ^3^ Department of Radiology Iiyama Red Cross Hospital Iiyama‐City Nagano‐ken Japan

**Keywords:** acetone, cerebrospinal fluid, phantom, relaxation time, T1 value, temperature

## Abstract

The T1 value of pure water, which is often used as a phantom to simulate cerebrospinal fluid, is significantly different from that of *in‐vivo* cerebrospinal fluid. The purpose of this study was to develop a phantom with a T1 value equivalent to that of *in‐vivo* cerebrospinal fluid under examination room temperature (23°C–25°C). In this study, 1.5 and 3.0 T magnetic resonance imaging scanners were used. We examined the signal intensity change in relation to pure water temperature, the T1 values of acetone‐diluted solutions (0–100 v/v%, in 10 steps), and the correlation coefficients obtained from volunteers and the prepared phantoms. The T1 value was close to the value reported in the literature for cerebrospinal fluid when the acetone‐diluted solution was 70 v/v% or higher at scan room temperature. The value at that time was 3532.81–4704.57 ms at 1.5 T and it ranged from 4052.41 to 5701.61 ms at 3.0 T. The highest correlation with the values obtained from the volunteers was *r* = 0.993 with pure acetone at 1.5 T and *r* = 0.991 with acetone 90 v/v% at 3.0 T. The relative error of the best phantom‐volunteer match was 32.61 (%) ± 6.71 at 1.5 T and 46.67 (%) ± 4.31 at 3.0 T. The T1 value measured by the null point method did not detect a significant difference between *in vivo* CSF and acetone 100 v/v% at 1.5 T and acetone 90 v/v% at 3.0 T. The T1 value of cerebrospinal fluid in the living body at scan room temperature was reproduced with acetone. The optimum concentration of acetone for cerebrospinal‐fluid reproduction was pure acetone at 1.5 T and 90 v/v% at 3.0 T.

## 1 INTRODUCTION

Validation using a phantom is important for magnetic resonance imaging (MRI) and other diagnostic imaging techniques. For example, MRI can be used to evaluate device performance, evaluate clinical‐image contrast, or optimize the sequence. Signal intensity in MRI varies with proton density, with T1, T2, and T2^*^ values; and with the diffusion coefficients inherent in tissues and lesions. The appropriate phantom to use differs based on the sequence to be verified and the phantom‐experiment purpose. Several reports exist on phantoms with the same T1 value, T2 value, diffusion coefficient, signal intensity, and morphology as the human‐body equivalent.^1^, ^2^, ^3^, ^4^, ^5^, ^6^ However, no report has reproduced very long T1 and T2 values, such as those found in cerebrospinal fluid (CSF).

Images in which the CSF is suppressed with normal MRI examination are clinically commonly used. T2‐fluid‐attenuated inversion recovery (FLAIR), T1‐FLAIR, and white matter attenuated inversion recovery (WAIR) using double inversion recovery (DIR) are clinically significant representative sequences.^7^, ^8^, ^9^, ^10^, ^11^, ^12^, ^13^, ^14^, ^15^, ^16^, ^17^, ^18^, ^19^, ^20^, ^21^, ^22^ The most important factor affecting contrast in these inversion recovery sequences is the T1 value. The substance most commonly used as a phantom to reproduce CSF is pure water (PW). PW is treated water with a reverse osmosis membrane to remove most of the organic matter and ion components. Moreover, it is known that suppression of CSF is insufficient in the FLAIR image in postmortem MRI examinations.^23^, ^24^, ^25^, ^26^, ^27^ This is probably because the T1 value changes due to the fact that the postmortem temperature is lower than the biological temperature. Tsukiashi et al. measured the T1 value against the water temperature and reported the change in the temperature and in the T1 value.^28^ From this, it is certain that the T1 value largely fluctuates due to changes in temperature. In addition, there is a possibility that the T1 value of PW under examination room temperature (RT) deviates markedly from that of *in‐vivo* CSF. Therefore, it is highly likely that CSF simulated with PW at RT cannot approximate the T1 value of the CSF in the living body. The difference in the T1 value between *in‐vivo* CSF and the PW phantom causes a large error when the imaging condition is changed, and therefore PW is unsuitable as a phantom to simulate CSF. Therefore, we attempted to develop a phantom able to reproduce the T1 value of CSF at RT.

## 2 MATERIALS AND METHODS

### 2.1 Materials

All images were acquired using a 3.0 T MRI scanner (Ingenia; Philips Healthcare, Amsterdam, Netherlands) and 1.5 T MRI scanner (Achieva d‐stream; Philips Healthcare). The solutions used were acetone (Matsuba, Ltd., Osaka, Japan) and PW (KENEI Pharmaceutical Co. Ltd., Osaka, Japan). A 10 mL (1.0 mL scale) graduated cylinder (AS ONE Co., Osaka, Japan) was used as the dispensing tool and an alcohol thermometer (Shinwa Rules Co., Niigata, Japan) was used for temperature measurement. The signal intensity was acquired using the ImageJ software (National Institutes of Health, Bethesda, MD).^29^

### 2.2 Dilution of acetone and the T1 value

Acetone was diluted with PW in 10 v/v% increments, and the T1 values were measured. The calculated values were obtained from the average of six measurements. The coefficient of variation (CV) of the six measurements was also calculated at the same time.

(1) $$\text{CV}=\frac{\text{Standard}\;\text{deviation}}{\text{Mean}\;\text{Signal}\;\text{Intensity}}\times 100\left(\%\right)$$

**Figure 1:**
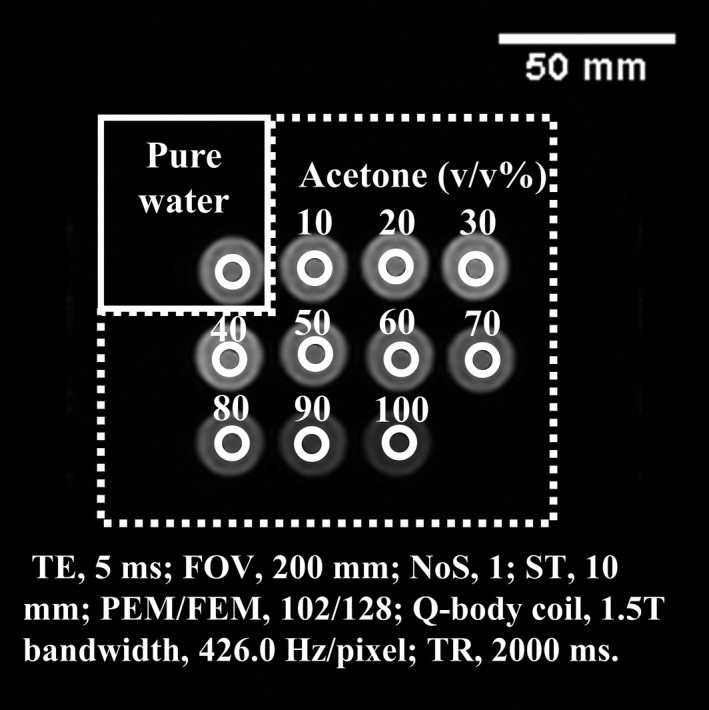
Placement of region of interests for T1 value measurement of the acetone dilution phantom. Magnetic resonance image of purified water and acetone dilutions (v/v%). A 7.02‐mm diameter ROI set in the pure water and acetone dilutions is shown.

The scan RT was between 23°C and 25°C. The saturation‐recovery method was used to measure the T1 value. The imaging parameters were as follows: TE, 5 ms; FOV, 200 mm; NoS, 1; ST, 10 mm; PEM/FEM, 102/128; Q‐body coil, and 1.5 T/3.0 T bandwidth, 426.0/602.4 Hz/pixel. The TR of the T1 measurement consisted of the following 21 points: 250, 500, 750, 1000, 2000, 4000, 6000, 8000, 10 000, 12 500, 15 000, 17 500, 20 000, 22 500, 25 000, 27 500, 30 000, 35 000, 40 000, 45 000, and 50 000 ms. Studies measuring the T1 value of CSF^30^, ^31^, ^32^, ^33^, ^34^, ^35^, ^36^ have reported values of 3817–6873 ms. From these values, the maximum TR was set to 50 000 ms so that the thermal equilibrium state recovered within the TR even when the T1 value was approximately 10 000 ms. The ROIs (7.02 mm in diameter) encompassing the PW and acetone 10–100 v/v% (10 v/v% increments; 10 points) were set (Fig. 1). The measurement was calculated using the nonlinear approximation of the least‐squares method using Excel 2016 Solver (Microsoft, Redmond, WA).

### 2.3 Comparison of the correlation and T1 value between the acetone Phantom and *In‐vivo* CSF

**Figure 2:**
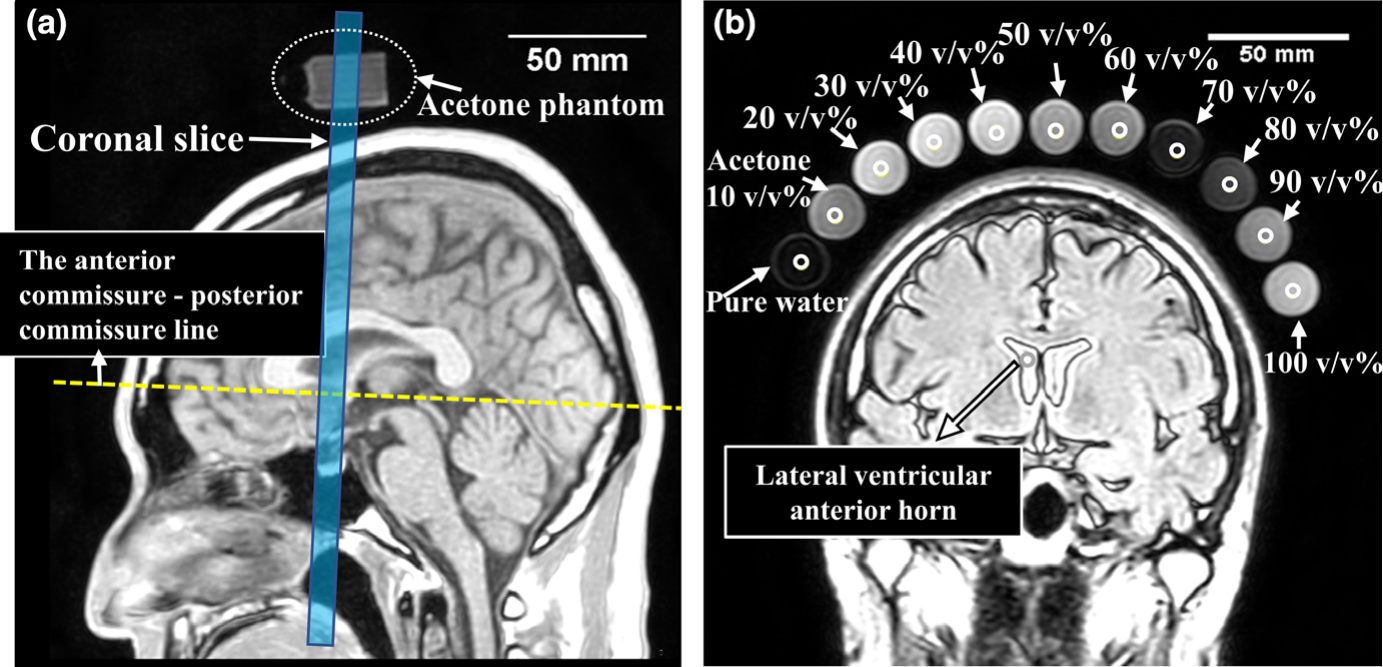
Reference line and region of interests set for correlation coefficient measurement of the acetone dilution phantoms and of the volunteers. (a) A healthy 35‐yr‐old man. Coronal sections of volunteers, pure water, and diluted acetone were obtained. The coronal section was arranged to cross the anterior commissure ‐ posterior commissure line; (b) the magnetic resonance image (TI 2000 ms) shows a 4.75‐mm diameter region of interest (white open circle) set in the lateral ventricular anterior horn, in pure water, and in acetone (10–100 v/v%).

The institutional ethics review board of our hospital approved this prospective study. Eight healthy volunteers responded to an open call from among staff working at our hospital. Informed consent was obtained from all volunteers. We aggregated eight individual adult studies (six men and two women; mean age, 36.7 yr; age range, 23–51 yr). Volunteers and solutions diluted with acetone placed on the head of volunteers were imaged at the same coronal section and images were obtained with TI change of the inversion recovery (IR) method [Fig. 2(a)]. The Spearman rank correlation coefficient between the signal intensity of CSF and the signal intensity of PW and acetone 60 to 100% was tested. All analyses were performed using SPSS (version 25.0, IBM Corp., Armonk, NY), and *P *< 0.001 were considered statistically significant. In addition, from the IR images with different TIs, the relative error between water and acetone 60 to 100 v/v% was calculated based on the *in‐vivo* CSF. In total, the mean of the relative error and standard deviation were calculated. The calculation formula of the relative error is set to Eq. (2).

(2) $$\text{Relative}\;\text{error}\;=\;\frac{\left(\text{Measured}\;\text{signal}\;\text{intensity}\;-\;\text{In - vivo}\;\text{CSF}\;\text{signal}\;\text{intensity}\right)}{\text{In - vivo}\;\text{CSF}\;\text{signal}\;\text{intensity}}\times 100\times \left(\%\right)$$

In addition, a null point calculation was added to compare the T1 values of biological CSF and the acetone‐water system. The calculation of the T1 value by the null point method was performed using Eq. (3).

(3) $$\text{T}1\text{value}=\frac{\text{T}1\text{null}}{\ln2}$$

Here, TInull is the TI time at which the signal intensity becomes 0.

The measurement result of the T1 value was tested between the biological CSF and PW and acetone 80–100 v/v% groups by Student's *t* test. The results were considered significant when *P *< 0.05.

The imaging parameters were as follows: TR/TE, 10,000/120 ms; FOV, 240 mm; NoS, 1; ST, 4 mm; PEM/FEM, 128/192; sensitivity encoding factor, 2; dS head/neck coil (20‐channel multi‐channel coil.), and 1.5/3.0 T bandwidth, 397.3/755.4 Hz/pixel. The TI varied in 250 ms increments from 500 to 5000 ms (19 values). The ROIs (4.75 mm in diameter) encompassing the lateral ventricular anterior horn, PW, and acetone 10–100 v/v% (10 v/v% increments; 10 points) were set [Fig. 2(b)]. Reversal of the longitudinal magnetization was provided by adiabatic pulses in the all studies. Moreover, SENCE was used by a factor of 2.0 for parallel imaging.

### 2.4 Long‐term change in relaxation time of the acetone water system

Changes in the T1 value in the acetone water system were obtained approximately 11 months after development and compared. The measurement was the same as that in method B, with the RT of 3.0 T at this time being 23°C and of 1.5 T being 24°C.

## 3 RESULTS

### 3.1 Dilution of acetone and T1 values

**Figure 3:**
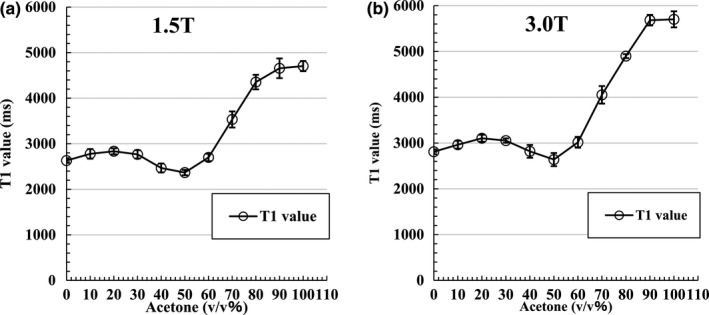
Measurement result of T1 value at acetone dilution. (a) Graph of T1 value by 1.5 T acetone dilution at 24°C ± 1°C. The T1 value is longer than that of PW when the acetone dilution is higher than 60 v/v%. The vertical lines and whiskers indicate the standard deviation; (b) graph of T1 value at 3.0 T with the acetone dilution at 24°C ± 1°C. The T1 value is longer than that of PW when the acetone dilution is higher than 60 v/v%. Vertical lines and whiskers show the standard deviation. Measurement points are connected by straight lines, not fitted curves.

Figure 3 shows the measurement results of T1 values by acetone dilution. The T1 value was larger for acetone than for PW. Additionally, the change in the T1 value was markedly prolonged at a dilution concentration of 60 v/v% or higher. The results obtained at dilution concentrations of 0 v/v% (PW) and 60 v/v% or higher are summarized in Table 1. Furthermore, values reported in the literature are summarized in Table 2.^30^, ^31^, ^32^, ^33^, ^34^, ^35^, ^36^ With the exception of 6873 ms reported by Liberman et al,^33^ we found that it was possible to obtain a T1 value similar to that of *in‐vivo* CSF with acetone at a dilution of 70–80 v/v% with 1.5 and 3.0‐T MRI. Moreover, the variation coefficient of the T1 value was measured six times at 23–25°C in the order of water and acetone 10–100 v/v%. At 1.5 T, it was 1.96, 3.82, 2.31, 3.31, 3.94, 2.50, 3.06, 5.01, 3.66, 4.63, 2.38 (%). At 3.0 T, it was 1.87, 2.89, 2.46, 1.59, 4.98, 5.43, 3.93, 4.72, 3.29, 2.04, 3.07 (%).

**Table 1:** T1 values obtained at acetone dilution concentrations of 0 v/v% (pure water) and ≥ 60 v/v%.

Acetone (v/v%)	0 (PW)	60	70	80	90	100
1.5 T	2631.32 (± 51.69)	2699.72 (± 82.51)	3532.81 (± 117.26)	4353.02 (± 159.42)	4656.63 (± 215.73)	4704.57 (± 112.13)
3.0 T	2809.63 (± 52.45)	3016.62 (± 118.70)	4052.41 (± 191.12)	4898.84 (± 161.40)	5683.28 (± 115.96)	5701.61 (± 175.19)

The data are presented as mean ms (±standard deviation).

**Table 2:** A summary of values in the literature for each magnetic‐field strength.

Author	Literature value (ms) 1.5 T	Literature value (ms) 3.0 T
Shin et al. 30	—	4391 ± 545 (IR‐EPI)
		4522 ± 417 (IR LL‐EPI‐SS)
Lu et al. 31	3836 ± 470	3817 ± 424
Chen et al. 32	—	4163 ± 263
Liberman et al. 33	—	6873 (women)
		4184 (men)
Helms et al. 34	—	4181
Rooney et al. 35	4070 ± 65	—
Steen et al. 36	4282	—

Abbreviations: IR‐EPI, inversion recovery echo‐planar imaging; IR LL‐EPI‐SS, inversion recovery Look‐Locker echo‐planar imaging at steady state.

### 3.2 Correlation between the acetone phantom and *In‐vivo* CSF

**Figure 4:**
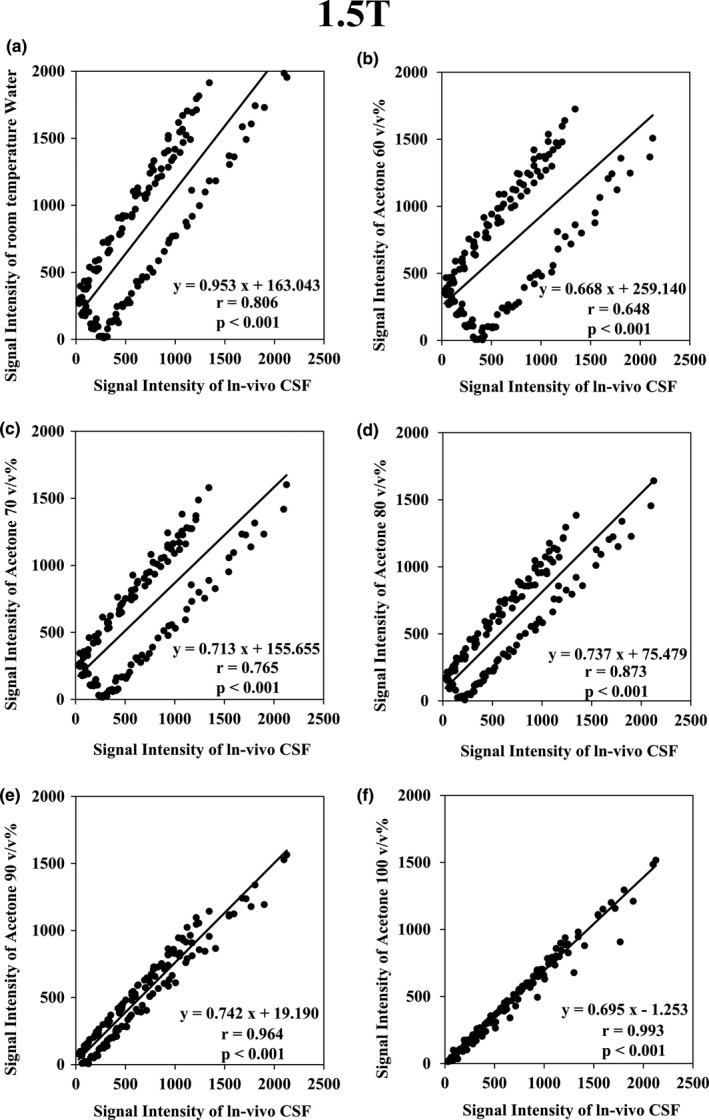
Graph of the Spearman rank correlation coefficient test results at 1.5 T. (a) Between *in‐vivo* cerebrospinal fluid (CSF) and purified water (*r *= 0.806; *P *< 0.001); (b) between *in‐vivo* CSF and acetone 60 v/v% (*r *= 0.648; *P *< 0.001); (c) between *in‐vivo* CSF and acetone 70 v/v% (*r *= 0.765; *P *< 0.001); (d) between *in‐vivo* CSF and acetone 80 v/v% (*r *= 0.873; *P *< 0.001); (e) between *in‐vivo* CSF and acetone 90 v/v% (*r *= 0.964; *P *< 0.001); f: Between *in‐vivo* CSF and acetone 100 v/v% (*r *= 0.993; *P *< 0.001).

**Figure 5:**
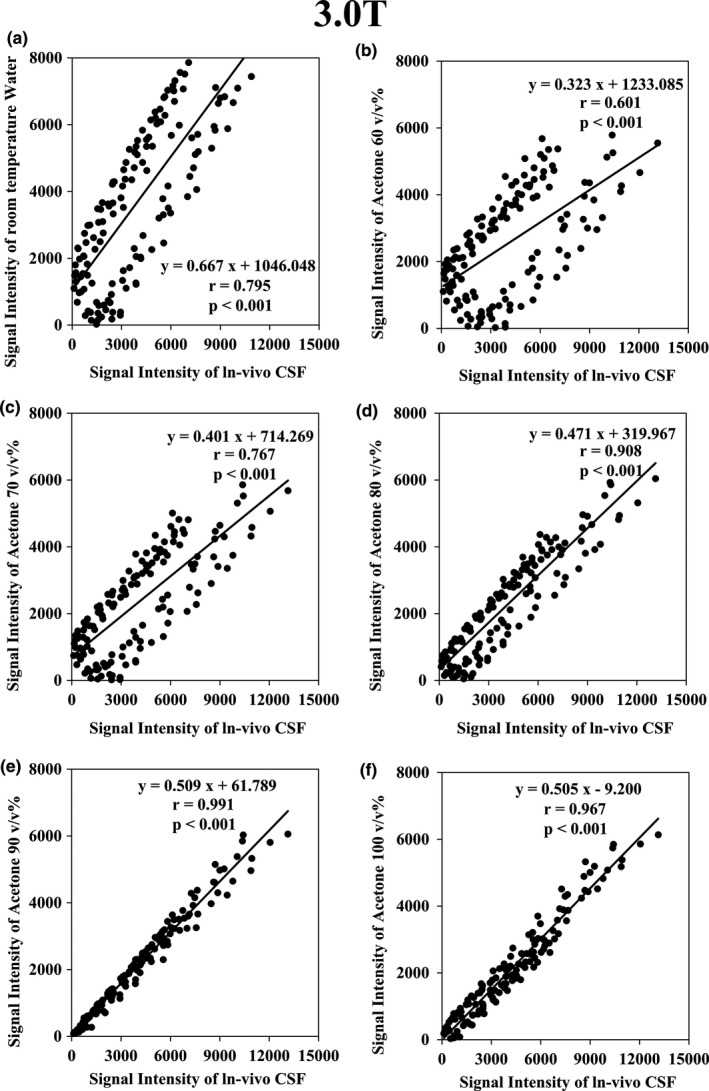
Graph of the Spearman rank correlation coefficient test results at 3.0 T. (a) Between *in‐vivo* cerebrospinal fluid (CSF) and purified water (*r *= 0.795; *P *< 0.001); (b) between *in‐vivo* CSF and acetone 60 v/v% (*r *= 0.601; *P *< 0.001); (c) between *in‐vivo* CSF and acetone 70 v/v% (*r *= 0.766; *P *< 0.001); (d) between *in‐vivo* CSF and acetone 80 v/v% (*r *= 0.908; *P *< 0.001); (e) Between *in‐vivo* CSF and acetone 90 v/v% (*r *= 0.991; *P *< 0.001); f: Between *in‐vivo* CSF and acetone 100 v/v% (*r *= 0.967; *P *< 0.001).

The results of the Spearman correlation with the signal intensity of PW and with the signal intensity of acetone at concentrations of 60, 70, 80, 90, and 100 v/v% are shown in Figs. 4 and 5. For all conditions (*P* < 0.001), the highest correlation with *in‐vivo* CSF was *r* = 0.993 with acetone 100 v/v% for 1.5 T and *r* = 0.991 with acetone 90 v/v% for 3.0 T.

**Figure 6:**
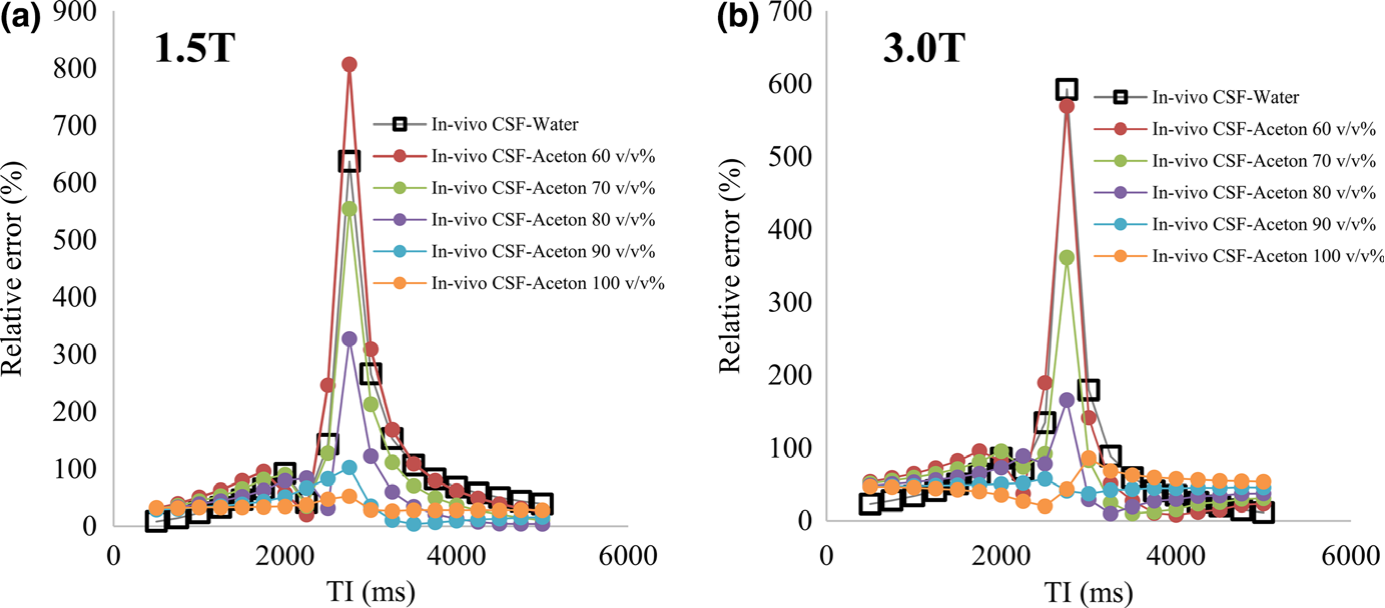
Relative error of pure water and acetone 60–100 v/v% at each TI based on *in‐vivo* cerebrospinal fluid. The vertical axis shows the relative error and the horizontal axis shows the TI. (a) Result at 1.5 T. (b) Result at 3.0 T.

The relative error of signal intensity at each TI between *in‐vivo* CSF, PW, and acetone 60–100 v/v% is shown in Fig. 6. On the image, there was a tendency for the error to become large near TI 2700 ms at which CSF became a null point. Especially, PW and acetone 60 v/v% were most prominent. In 1.5 T, acetone 100 v/v% and in 3.0 T, acetone 90 v/v% had a small relative error and flat change.

**Figure 7:**
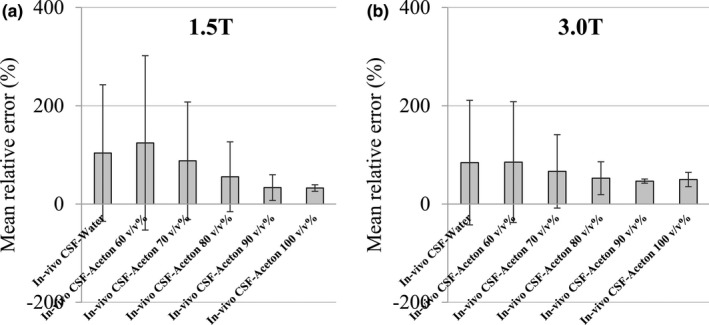
Average relative error of pure water and acetone 60–100 v/v% based on *in‐vivo* cerebrospinal fluid (CSF). The vertical axis is the average relative error and the horizontal axis is the sample concentration based on the *in‐vivo* CSF. Vertical lines and whiskers show the standard deviation. (a) Result at 1.5 T. (b) Result at 3.0 T.

The results of averaging the relative errors are shown in Fig. 7. At 1.5 T, 103.89 (%) ± 138.95 at PW, 124.49 (%) ± 177.42 at acetone 60 v/v%, 88.09 ± 119.62 at acetone 70 v/v%, 55.53 (%) ± 70.99 at acetone 80 v/v%, 33.48 (%) ± 26.21 at acetone 90 v/v%, 32.61 (%) ± 6.71 at acetone 100 v/v%, with that of pure acetone being the lowest. At 3.0 T, 84.31 (%) ± 126.81 at PW, 85.26 (%) ± 123.00 at acetone 60 v/v%, 66.54 ± 74.78 at acetone 70 v/v%, 52.61 (%) ± 33.44 at acetone 80 v/v%, 46.67 (%) ± 4.31 at acetone 90 v/v%, 49.87 (%) ± 14.50 at acetone 100 v/v%, with that of acetone 90 v/v% being the lowest.

Figure 9 shows the results of the T1 values measured using the null point method. At 1.5 T, the T1 value of *in‐vivo* CSF was 3831.54 ± 105.46 ms, and the values of acetone 80–100 v/v% are presented below. The value was 3159.17 ± 66.06 at acetone 80 v/v%, 3535.82 ± 54.24 at acetone 90 v/v%, and 3939.91 ± 124.49 ms at 100 acetone v/v%. The PW T1 value was 2935.98 ± 56.26 ms. According to the results of Student's *t* test performed between the *in‐vivo* CSF and PW and acetone 80–100 v/v% groups, the difference between the *in‐vivo* CSF and PW and acetone 80–90 v/v% was significant at *P* < 0.05. There was no significant difference between *in‐vivo* CSF and acetone 100 v/v% at *P* = 0.0786. At 3.0 T, the *in‐vivo* CSF T1 value was 3970.07 ± 115.60 ms, that of acetone 80 v/v% was 3337.89 ± 84.68, of acetone 90 v/v% was 387.23 ± 99.55, and of acetone 100 v/v% was 4141.79 ± 109.19 ms. The PW T1 value was 3002.87 ± 66.83 ms. In Student's *t* test, there was a significant difference at *P* < 0.05 between *in‐vivo* CSF and PW, acetone 80 v/v%, and acetone 100 v/v%, but there was no significant difference between *in‐vivo* CSF and acetone 90 v/v% at *P* = 0.145.

### 3.3 Long‐term change in relaxation time of the acetone water system

**Figure 8:**
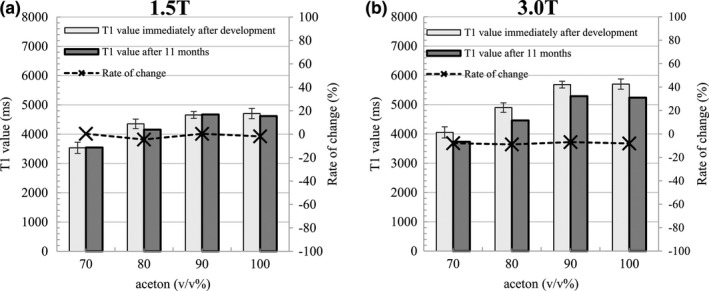
T1 value of acetone 70–100 v/v% obtained approximately 11 months after development. The left vertical axis shows the T1 value and the horizontal axis shows the acetone concentration. The right vertical axis corresponds to the dotted line in the rate of change. Vertical lines and whiskers show the standard deviation. (a) Result at 1.5 T. (b) Result at 3.0 T.

Figure 8 shows the change in the T1 value of acetone 70–100 v/v% obtained approximately 11 months after development. At 1.5 T, there was no major change in the T1 value, and the rate of change remained within 5%. There was no marked change in 3.0 T either, and the change rate was approximately 8% at the maximum.

**Figure 9:**
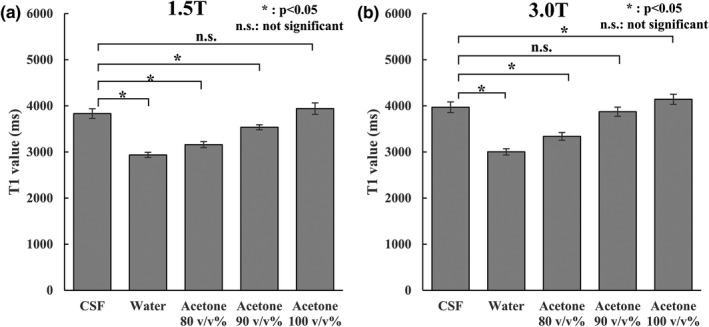
Results of the T1 value calculations using the null point method. The vertical axis shows the T1 value, and the horizontal axis shows the sample. *P < 0.05, and n.s. indicates no significant difference. Vertical lines and whiskers show the standard deviation. (a) Result at 1.5 T. (b) Result at 3.0 T.

## 4 DISCUSSION

Here, we developed a phantom that reproduced the T1 value of CSF at RT (23°C–25°C). PW at RT has a much lower T1value than that of *in‐vivo* CSF and thus is not suitable as a phantom for CSF reproduction. We solved this problem using acetone.

It is well known that the T1 value varies with temperature.^28^ Therefore, by constructing a system that can be adjusted so that the phantom temperature can be maintained at a level a researcher desires, it is possible to develop a phantom that has a similar T1 value as that of *in‐vivo* CSF. However, the thermoregulatory equipment cannot be used in many facilities. Acetone‐water mixtures can match *in‐vivo* the T1 of CSF without additional thermoregulatory equipment.

The T1 value measurement results revealed that acetone had a T1 value much higher than that of PW at RT. Therefore, we believed that the same T1 value as that of *in‐vivo* CSF could be reproduced at RT by diluting acetone with PW. To date, the relaxation of water‐acetone series has not been well studied. Therefore, it remains unclear why the T1 value of acetone is longer than that of PW. However, we assume that the cause may be the difference in chemical shift with PW. In the scarce available literature, there is a statement on chemical shifts related to water and acetone series.^37^ According to that study, it was inferred that the difference in the peak of the water‐acetone system is approximately 2.4 ppm.^37^ That is, the chemical shift of acetone is larger than that of water. It is known that the rate of relaxation based on the mechanism of the magnetic resonance phenomenon slows as the proportion of spins belonging to the resonance frequency band decreases in the Larmor frequency distribution, resulting in a longer T1 value.^38^. Therefore, it was considered that the resonance frequency of acetone became dominant as the concentration of acetone increased in the water‐acetone system, and the T1 value increased. Acetone is a type of ketone represented by the symbolic formula CH3OCH3. Electron‐rich nucleophiles attack this ketone because the carbonyl group (CO) it possesses is positively charged. As a result, the π electron of the C = O bond moves to the oxygen atom, and the oxygen atom acquires a formal negative charge. If these reactions are carried out in a solvent with a hydroxy group (−OH), such as H2O, a proton H+ will usually be added to this negative charge. This reaction is termed "nucleophilic addition" and is considered to occur in a mixture of acetone and water. There is reported oxygen molecules in acetone form hydrogen bonds with surrounding water molecules and interact strongly with each other.^39^ However, the linewidth of acetone and water is narrow based on the spectral peak reported in the literature^36^; it is expected to have a relatively slow chemical exchange rate, as it is observed as an independent peak. If the exchange between those two pools is slow, the acetone protons and water protons may retain their own relaxation time to certain degree. Therefore, it should be noted that there is a possibility that T1 relaxation cannot be accurately characterized with a single exponential function.

A T1 value close to those reported in the literature^30^, ^31^, ^32^, ^33^, ^34^, ^35^, ^36^ could be obtained with a 70–80 v/v% dilution of acetone at 1.5 T and 3.0 T. Although these measurements are very useful, they are not specialized in measuring long T1 values such as that of CSF. For this reason, the variation was large in these previous measurements of the T1 value of CSF. As stated by Liberman et al,^33^ this fluctuation arises from flip angle (FA) inaccuracy, B1 inhomogeneity, and the partial volume effect of the ROI setting and also depends on the method, the number of measurement samples, and the patient group. We were concerned that the ghosting artifact would influence the calculation result of the T1 value by arranging the signal substance in the phase direction. However, since it was confirmed that there was no influence in the experiment at the previous stage, we selected to place the phantom as close to the center of the magnetic field as possible. In order to reduce magnetic susceptibility artifacts, the surroundings of the phantom often satisfy some type of filling material, but signal unevenness due to B1 inhomogeneity occurred; hence, in this study, the influence of B1 inhomogeneity was eliminated to the greatest possible extent without using filling material. Furthermore, we selected a method to change the TR to more accurately measure a long T1 value as that of CSF. In this method, the inaccuracy of IR due to B1 inhomogeneity and the measurement error due to TI setting limitation are relaxed. The saturation recovery method used a 90° pulse to reverse longitudinal magnetization. In this method, the FA is fixed, and the T1 value is calculated from the data obtained by changing the TR. Therefore, the error of the T1 value calculated when the FA is inaccurate was numerically simulated. Even if the 90° pulse was changed to 50°–130°, the calculated T1 value had an error of 1% or lower. However, the proton density was calculated to deviate as the error of FA increased. This was considered to be because the error of FA affected the signal strength, but the error was the same for each TR image and did not significantly affect the relaxation time calculated by nonlinear fitting. For this reason, although B1 nonuniformity was not corrected, it is considered that the T1 value was calculated with sufficient accuracy.

Figures 4 and 5 show that the signal intensity of the diluted solution of acetone is lower than that of *in‐vivo* CSF. This is probably due to the difference in proton density. However, the correlation of the signal intensity with changing TI increased with the increase in acetone concentration. This signifies that the signal intensity scales differ, but the signal changes are very similar. To wit, it denotes that the T1 value of *in‐vivo* CSF is very similar to that of acetone and acetone diluted in water. Therefore, we provided evidence that the T1 value of *in‐vivo* CSF can be reproduced using acetone. Our T1 measurements had RT variations of 23–25°C. However, the coefficient of variation remained within approximately 5% in these measurements. This indicates that the change in the T1 value due to the temperature of the acetone water system in the range of 23 to 25°C, as measured here, is very small. The MRI systems of most facilities should be maintained at RTs of approximately 23°C. Therefore, our data should be applicable to most facilities.

Furthermore, from Fig. 6, we observed a large difference in the relative error near the null point in the FLAIR image. For example, it is possible that the image using IR may show a large difference between an inappropriate phantom and *in‐vivo* CSF. Studies utilizing tissue volumes are actively conducted with voxel‐based morphometry of brain tissue.^40^, ^41^, ^42^, ^43^ In order to enhance the contrast between white matter and gray matter, the IR pulse may be used in advance. In this case, when performing a quantitative phantom experiment, the signal strength of CSF may be different from that of the living body. There is a possibility that the signal difference from the *in‐vivo* CSF becomes larger, which may be misrecognized as white matter or gray matter. In addition, imaging using IR (T2 — FLAIR, T1 — FLAIR, or WAIR) is expected to greatly influence the suppression of CSF, and the influence of the difference in the T1 value on the verification result is concerning. As shown in Fig. 8, the relative error with acetone 60–100 v/v% compared with the *in vivo* CSF at 1.5 T was 124.49 (%) ± 177.42 to 32.61 (%) ± 6.71. At 3.0 T, it varied from 85.26 (%) ± 123.00 to 49.87 (%) ± 14.50, and the minimum value was 46.67 (%) ± 4.31 at acetone 90 v/v%. From this, it is considered important to select an appropriate acetone concentration when conducting a quantitative verification using a phantom.

Finally, it was important to directly compare the actual *in‐vivo* CSF and acetone‐water system T1 values, and the measurement by the null point method was added. This is a relatively simple measurement method of calculating the T1 value from the TI time when the signal strength is zero. In this measurement condition, when the measurement target has a long T1 value, as is the case for *in‐vivo* CSF because the TR is 10 000 ms, it is highly possible that longitudinal magnetization is not completely recovered within the TR time. For this reason, the calculated T1 value is considered underestimated. Given the condition where TR is 10 000 ms, it is considered that the T1 value can be measured accurately up to approximately 2000 ms, but based on the result of the saturation recovery method, all measurement targets of *in‐vivo* CSF and the acetone‐water system were higher than 2000 ms. Therefore, the T1 values were underestimated, but it is considered that the same error occurred in the *in‐vivo* CSF and phantom groups and may be appropriate for the comparison of the T1 values. In this comparison, there was no significant difference between *in‐vivo* CSF and acetone 100 v/v% at 1.5 T and acetone 90 v/v% at 3.0 T and was in line with the previous verification results. From the above, it is clear that PW at RT cannot reproduce the T1 value of *in‐vivo* CSF, and acetone 100 v/v% at 1.5 T was appropriate to reproduce the T1 value of *in‐vivo* CSF under RT (23–25°C). The appropriate selection at 3.0 T was shown to be acetone 90 v/v%.

The T1 value of the acetone‐water system observed for long‐term fluctuation did not vary greatly. This suggesting that stable use is possible in the long‐term.

It is hence possible to reproduce the T1 value of *in‐vivo* CSF using acetone, which cannot be performed using PW. We believe that our findings may contribute to MRI research focusing on the CSF.

The purpose of this study was to reproduce the T1 value of CSF at RT. As a limiting factor, the proton density and T2 value may have been imperfect.

## 5 CONCLUSION

The CSF T1 value in the living body at scanner RT was reproduced with acetone. The optimum acetone concentration that reproduced the CSF T1 value was pure acetone at 1.5 T and acetone 90 v/v% at 3.0 T.

The findings of this study may be useful for MRI studies that focus on the brain.

## CONFLICT OF INTEREST

The authors declare no conflict of interest.

## References

[1] Mitchell MD , Kundel HL , Axel L , Joseph PM . Agarose as a tissue equivalent phantom material for NMR imaging. Magn Reson Imaging. 1986;4:263–266. 3669940 10.1016/0730-725x(86)91068-4

[2] Yoshimura K , Kato H , Kuroda M , et al. Development of a tissue‐equivalent MRI phantom using carrageenan gel. Magn Reson Med. 2003;50:1011–1017. 14587012 10.1002/mrm.10619

[3] Kato H , Kuroda M , Yoshimura K , et al. Composition of MRI phantom equivalent to human tissues. Med Phys. 2005;32:3199–3208. 16279073 10.1118/1.2047807

[4] Ikemoto Y , Takao W , Yoshitomi K , et al. Development of a human‐tissue‐like phantom for 3.0‐T MRI. Med Phys. 2011;38:6336–6342. 22047398 10.1118/1.3656077

[5] Laubach HJ , Jakob PM , Loevblad KO , et al. A phantom for diffusion‐weighted imaging of acute stroke. J Magn Reson Imaging. 1998;8:1349–1354. 9848751 10.1002/jmri.1880080627

[6] Saotome K , Matsushita A , Matsumoto K , et al. A brain phantom for motion‐corrected PROPELLER showing image contrast and construction similar to those of in vivo MRI. Magn Reson Imaging. 2017;36:32–39. 27742431 10.1016/j.mri.2016.10.003

[7] Kuwazuru J , Arimura H , Kakeda S , et al. Automated detection of multiple sclerosis candidate regions in MR images: false‐positive removal with use of an ANN‐controlled level‐set method. Radiol Phys Technol. 2012;5:105–113. 22139608 10.1007/s12194-011-0141-2

[8] Yuan MK , Lai PH , Chen JY , et al. Detection of subarachnoid hemorrhage at acute and subacute/chronic stages: comparison of four magnetic resonance imaging pulse sequences and computed tomography. J Chin Med Assoc. 2005;68:131–137. 15813247 10.1016/S1726-4901(09)70234-5

[9] Morais DF , Spotti AR , Tognola WA , Gaia FF , Andrade AF . Clinical application of magnetic resonance in acute traumatic brain injury. Arq Neuropsiquiatr. 2008;66:53–58. 18392415 10.1590/s0004-282x2008000100013

[10] Jack CR Jr , Rydberg CH , Krecke KN , et al. Mesial temporal sclerosis: diagnosis with fluid‐attenuated inversion‐recovery versus spin‐echo MR imaging. Radiology. 1996;199:367–373. 8668780 10.1148/radiology.199.2.8668780

[11] Splendiani A , Puglielli E , De Amicis R , Necozione S , Masciocchi C , Gallucci M . Contrast‐enhanced FLAIR in the early diagnosis of infectious meningitis. Neuroradiology. 2005;47:591–598. 16034600 10.1007/s00234-005-1383-7

[12] Kamran S , Bener AB , Alper D , Bakshi R . Role of fluid‐attenuated inversion recovery in the diagnosis of meningitis: comparison with contrast‐enhanced magnetic resonance imaging. J Comput Assist Tomogr. 2004;28:68–72. 14716235 10.1097/00004728-200401000-00011

[13] Vaswani AK , Nizamani WM , Ali M , Aneel G , Shahani BK , Hussain S . Diagnostic accuracy of contrast‐enhanced FLAIR magnetic resonance imaging in diagnosis of meningitis correlated with CSF analysis. ISRN Radiol. 2014;2014:578986. 24977138 10.1155/2014/578986 PMC4062848

[14] Galassi W , Phuttharak W , Hesselink JR , Healy JF , Dietrich RB , Imbesi SG . Intracranial meningeal disease: comparison of contrast‐enhanced MR imaging with fluid‐attenuated inversion recovery and fat‐suppressed T1‐weighted sequences. AJNR Am J Neuroradiol. 2005;26:553–559. 15760865 PMC7976502

[15] Wattjes MP , Lutterbey GG , Gieseke J , et al. Double inversion recovery brain imaging at 3T: diagnostic value in the detection of multiple sclerosis lesions. AJNR Am J Neuroradiol. 2007;28:54–59. 17213424 PMC8134107

[16] Seewann A , Kooi EJ , Roosendaal SD , et al. Postmortem verification of MS cortical lesion detection with 3D DIR. Neurology. 2012;78:302–308. 22218278 10.1212/WNL.0b013e31824528a0

[17] Kolber P , Montag S , Fleischer V , et al. Identification of cortical lesions using DIR and FLAIR in early stages of multiple sclerosis. J Neurol. 2015;262:1473–1482. 25862481 10.1007/s00415-015-7724-5

[18] van de Pavert SH , Muhlert N , Sethi V , et al. DIR‐visible grey matter lesions and atrophy in multiple sclerosis: partners in crime? J Neurol Neurosurg Psychiatry. 2016;87:461–467. 25926483 10.1136/jnnp-2014-310142 PMC4853554

[19] Oda S , Shimoda M , Hirayama A , et al. Neuroradiologic diagnosis of minor leak prior to major SAH: diagnosis by T1‐FLAIR mismatch. AJNR Am J Neuroradiol. 2015;36:1616–1622. 25977479 10.3174/ajnr.A4325 PMC7968783

[20] Wang G , Wang J , Zhan J , et al. Quantitative assessment of cerebral gray matter density change in progressive supranuclear palsy using voxel based morphometry analysis and cerebral MR T1‐weighted FLAIR imaging. J Neurol Sci. 2015;359:367–372. 26671144 10.1016/j.jns.2015.11.007

[21] Downs RK , Bashir MH , Ng CK , Heidenreich JO . Quantitative contrast ratio comparison between T1 (TSE at 1.5 T, FLAIR at 3 T), magnetization prepared rapid gradient echo and subtraction imaging at 1.5 T and 3 T. Quant Imaging Med Surg. 2013;3:141–146. 23833727 10.3978/j.issn.2223-4292.2013.05.02 PMC3701100

[22] Ganesan K , Bydder GM . A prospective comparison study of fast T1 weighted fluid attenuation inversion recovery and T1 weighted turbo spin echo sequence at 3 T in degenerative disease of the cervical spine. Br J Radiol. 2014;87:20140091. 25010068 10.1259/bjr.20140091 PMC4453141

[23] Kobayashi T , Shiotani S , Kaga K , et al. Characteristic signal intensity changes on postmortem magnetic resonance imaging of the brain. JPN J Radiol. 2010;28:8–14. 20112087 10.1007/s11604-009-0373-9

[24] Abe K , Kobayashi T , Shiotani S , et al. Optimization of inversion time for postmortem fluid‐attenuated inversion recovery (FLAIR) MR imaging at 1.5 T: temperature‐based suppression of cerebrospinal fluid. Magn Reson Med Sci. 2015;14:251–255. 25833274 10.2463/mrms.2014-0086

[25] Kobayashi T , Isobe T , Shiotani S , et al. Postmortem magnetic resonance imaging dealing with low temperature objects. Magn Reson Med Sci. 2010;9:101–108. 20885082 10.2463/mrms.9.101

[26] Ruder TD , Thali MJ , Hatch GM . Essentials of forensic post‐mortem MR imaging in adults. Br J Radiol. 2014;87:20130567. 24191122 10.1259/bjr.20130567 PMC4067017

[27] Tofts PS , Jackson JS , Tozer DJ , et al. Imaging cadavers: cold FLAIR and noninvasive brain thermometry using CSF diffusion. Magn Reson Med. 2008;59:190–195. 18058937 10.1002/mrm.21456 PMC2478723

[28] Tsukiashi A , Min Kil Sik , Kitayama H , et al. Application of spin‐crossover water soluble nanoparticles for use as MRI contrast agents. Sci Rep. 2018;8:14911. 30297794 10.1038/s41598-018-33362-6 PMC6175957

[29] Rasband W . ImageJ, U.S. National Institutes of Health. https://rsb.info.nih.gov/ij, 1997‐2012. Accessed May 30, 2018.

[30] Shin W , Gu H , Yang Y . Fast high‐resolution T1 mapping using inversion‐recovery Look‐Locker echo‐planar imaging at steady state: optimization for accuracy and reliability. Magn Reson Med. 2009;61:899–906. 19195021 10.1002/mrm.21836 PMC2663603

[31] Lu H , Nagae‐Poetscher LM , Golay X , Lin D , Pomper M , vanZijl PC . Routine clinical brain MRI sequences for use at 3.0 Tesla. J Magn Reson Imaging. 2005;22:13–22. 15971174 10.1002/jmri.20356

[32] Chen L , Bernstein M , Huston J , Fain S . Measurements of T1 relaxation times at 3.0 T: implications for clinical MRA. In: Proceedings the 9th Annual Meeting of the International Society for Magnetic Resonance Medicine (Glasgow, Scotland). Berkeley, CA: International Society for Magnetic Resonance in Medicine; 2001:1.

[33] Liberman G , Louzoun Y , Ben BD . T_1_ mapping using variable flip angle SPGR data with flip angle correction. J Magn Reson Imaging. 2014;40:171–180. 24990618 10.1002/jmri.24373

[34] Helms G , Dathe H , Kallenberg K , Dechent P . High‐resolution maps of magnetization transfer with inherent correction for RF inhomogeneity and T1 relaxation obtained from 3D FLASH MRI. Magn Reson Med. 2008;60:1396–1407. 19025906 10.1002/mrm.21732

[35] Rooney WD , Johnson G , Li X , et al. Magnetic field and tissue dependencies of human brain longitudinal 1H2O relaxation in vivo. Magn Reson Med. 2007;57:308–318. 17260370 10.1002/mrm.21122

[36] Steen RG , Gronemeyer SA , Kingsley PB , Reddick WE , Langston JS , Taylor JS . Precise and accurate measurement of proton T1 in human brain in vivo: validation and preliminary clinical application. J Magn Reson Imaging. 1994;4:681–691. 7981513 10.1002/jmri.1880040511

[37] Hu HH , Nayak KS . Change in the proton T1 of fat and water in mixture. Magn Reson Med. 2010;63:494–501. 19918888 10.1002/mrm.22205 PMC2813964

[38] Koenig SH , Brown RD 3rd . Determinants of proton relaxation rates in tissue. Magn Reson Med. 1984;1:437–449. 6100933 10.1002/mrm.1910010404

[39] Takebayashi Y , Otake K . Molecular interactions and rotational dynamics in clathrate hydrate. Rev High Press Sci Technol. 2002;12:22–27.

[40] Matsuda H . Volumetry of cerebral gray and white matter using VSRAD®. Brain Nerve. 2015;67:487–496. 25846597 10.11477/mf.1416200165

[41] Carceller‐Sindreu M , Serra‐Blasco M , de Diego‐Adeliño J , et al. Altered white matter volumes in first‐episode depression: evidence from cross‐sectional and longitudinal voxel‐based analyses. J Affect Disord. 2019;15:971–977. 10.1016/j.jad.2018.11.085 30699883

[42] Long Z , Huang J , Li B , et al. A comparative atlas‐based recognition of mild cognitive impairment with voxel‐based morphometry. Front Neurosci. 2018;6:916. 10.3389/fnins.2018.00916 PMC6291519 30574064

[43] Lan DY , Zhu PW , He Y , et al. matter volume changes in patients with acute eye pain: a voxel‐based morphometry study. *Transl Vis* . Sci Technol. 2019;8:1. 10.1167/tvst.8.1.1 PMC6322711 30627476

